# Comparative Alignment of CFTR2 and CFTR-France Classifications with a Turkish Cystic Fibrosis Referral Cohort

**DOI:** 10.3390/ijms27177715

**Published:** 2026-08-28

**Authors:** Mustafa Tarık Alay, Filiz Özdemir, Deniz Agirbasli, Mehmet Seven, Aysel Kalayci

**Affiliations:** 1 Department of Medical Genetics, Ankara Etlik City Hospital, 06170 Ankara, Türkiye; mtarikalay@gmail.com; 2 Department of Medical Genetics, Cerrahpaşa School of Medicine, Istanbul University-Cerrahpaşa, 34098 Istanbul, Türkiye; flzozdmr@gmail.com (F.Ö.); deniz.agirbasli@iuc.edu.tr (D.A.); mimseven@gmail.com (M.S.)

**Keywords:** CFTR, cystic fibrosis, CFTR2, CFTR-France, variant classification, database comparison

## Abstract

*CFTR* variant spectra vary across populations, potentially limiting the transferability of reference classifications. We compared CFTR2 and CFTR-France with a single-centre Turkish cystic fibrosis referral cohort of 418 individuals tested between 2016 and 2022. To avoid incorporation bias, the primary analysis used measured sweat-test status as a genetics-independent anchor; unperformed tests were excluded rather than counted as negative. A *CFTR* variant was detected in 162/418 individuals (38.8%). Sweat-test results were available for 148 individuals: 85 positive and 63 negative. Variant detection was more frequent among sweat-positive individuals (50/85 vs. 23/63; OR 2.48, 95% CI 1.27–4.86; Fisher’s exact *p* = 0.008). Excluding exact ties, measured-sweat alignment was 22/28 (78.6%, 95% CI 59.0–91.7%) for CFTR2 and 15/21 (71.4%, 95% CI 47.8–88.7%) for CFTR-France. Direct database agreement was 362/395 (91.6%; Cohen’s κ = 0.74) across three classes and 330/330 (100%) for directional binary labels. CFTR2 and CFTR-France represented 64/75 (85.3%) and 58/75 (77.3%) cohort variants, respectively. Measured-sweat alignment was high for CF-causing but variable for non-CF-causing variants. Therefore, recurrent non-CF-causing variants should be reviewed case by case; the present data alone do not justify pathogenic reclassification, and phase-resolved and functional studies are required.

## 1. Introduction

Cystic fibrosis (CF) is a multisystem disorder resulting from the dysfunction of the cystic fibrosis transmembrane conductance regulator (CFTR) protein, characterized by progressive pulmonary deterioration and exocrine pancreatic insufficiency. It is the most common life-limiting autosomal recessive disorder in populations of European descent and is caused by biallelic pathogenic variants in the *CFTR* gene located on chromosome 7q31.2 [1]. The *CFTR* gene spans approximately 230 kb on chromosome 7q31.2, comprises 27 exons and 26 introns, and encodes a 1480-amino-acid epithelial chloride and bicarbonate channel [2]. Alternative splicing can alter CFTR transcript composition, including exon 10 skipping (historically termed exon 9 skipping) [3].

The frequency of observed CF disease varies markedly with geography and ancestry, affecting roughly one in 2000–3000 newborns in Europe and about one in 3500 in North America, whereas in Central Anatolia it is estimated at approximately one in 3400, despite a reported consanguinity rate of 20–25%. Although the management of CF has improved considerably over time, it remains a major cause of morbidity and mortality [4]. However, with correct modulation of the disease, many complications related to lung function, nutrition, and extrapulmonary outcomes can be prevented. Following the pivotal 2011 trial and the clinical introduction of ivacaftor in 2012, CFTR modulators acting directly on the underlying protein defect have substantially altered the clinical course and prognosis of cystic fibrosis [5,6]. Appropriate management begins with early diagnosis [2,7,8]. Newborn screening programmes, now implemented in many countries, have been repeatedly associated with better nutritional and pulmonary outcomes and improved survival, underlining that the benefits of modern therapy can only be fully realized when the diagnosis is made early and accurately [9,10].

In the molecular era, although CF can still be diagnosed based on clinical features and sweat testing, a confident diagnosis of CF is rarely established without careful molecular evaluation. Overall, 2092 *CFTR* gene variants have been catalogued in CFTR2 [https://cftr2.org], and their frequencies differ substantially between ethnic and geographic groups [11,12]. This diversity is particularly pronounced in Türkiye: whereas F508del accounts for roughly 70% of alleles in Northern Europe and North America, it represents fewer than 50% of alleles in the Mediterranean and only about 30% in Turkish patients. The Turkish population displays one of the highest degrees of allelic heterogeneity at the *CFTR* locus described so far [4]. Consequently, pre-set mutation panels designed for European populations perform poorly in Turkish patients, and comprehensive sequencing of the coding region and flanking intron boundaries is required to reach acceptable detection rates [4,13]. However, with the advancement of next-generation sequencing (NGS) technologies, a major challenge is the interpretation of variants of uncertain significance [14,15,16]. Moreover, a given variant may be classified differently from one population to another and may be associated with different clinical outcomes [17].

Locus-specific databases such as the Clinical and Functional Translation of CFTR (CFTR2) project and CFTR-France were established to characterize the clinical impact and phenotypic spectrum of *CFTR* gene variants, yet population-genetics information is still incorporated to only a limited extent and the penetrance of many CFTR variants remains poorly defined [11,18,19]. Because the spectrum, frequency and even the phenotypic consequences of *CFTR* gene variants vary with ethnic and geographic background, characterizing and comparing variant data across populations is essential for accurate molecular diagnosis, reliable carrier testing and appropriate genotype-based counselling [20,21]. Türkiye, with its high consanguinity, marked allelic heterogeneity and comparatively low F508del frequency, provides a genetic landscape that differs considerably from that of Western European cohorts, making it informative for cross-dataset comparison [13,22]. In the present study, normalized Turkish cohort variants were compared independently with the CFTR-France all-variant export dated 6 October 2025 and the CFTR2 release dated 30 January 2026; we also quantified direct agreement between the two resources and their alignment with the genetics-independent measured-sweat endpoint.

## 2. Results

**Figure 1:**
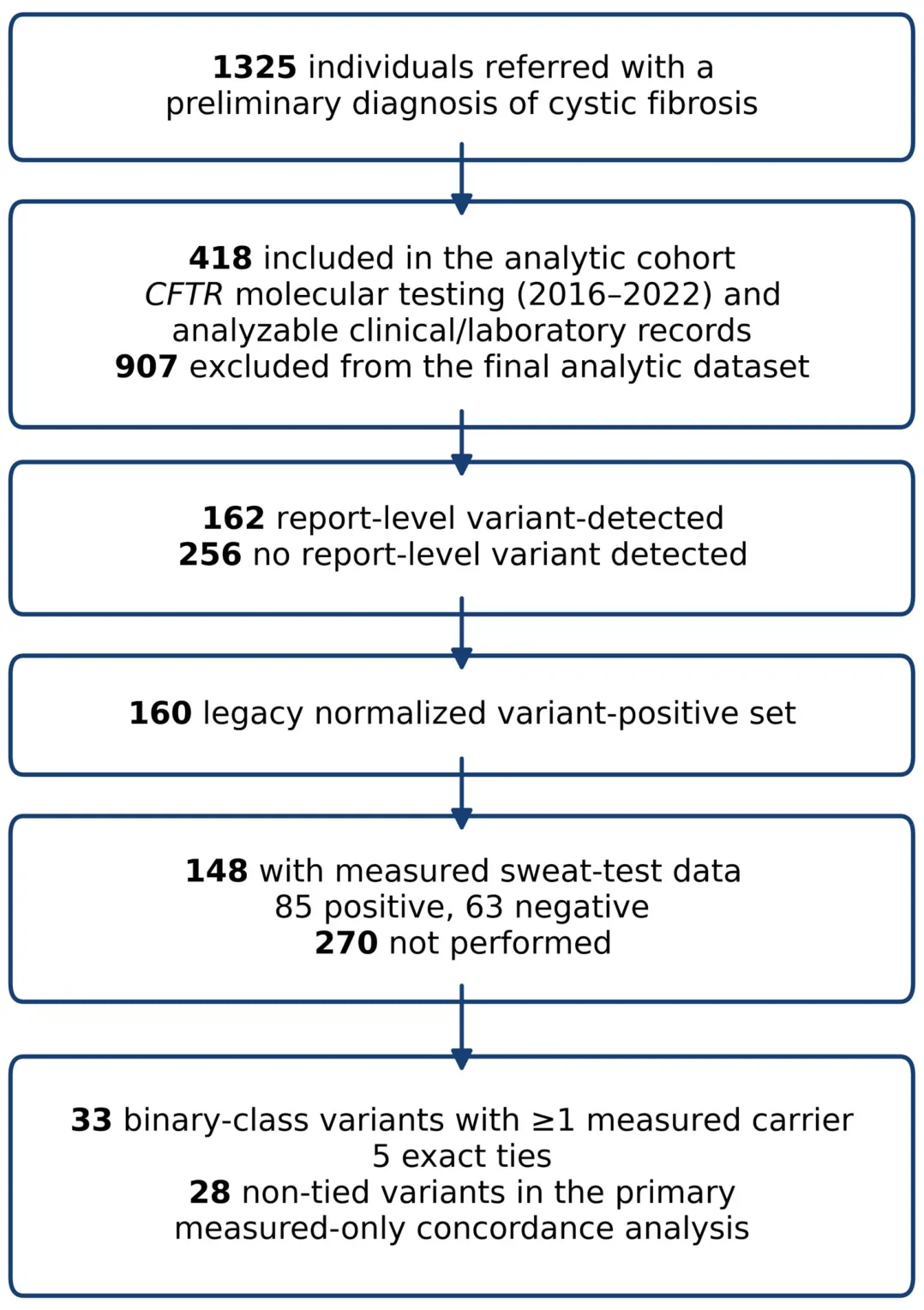
Flowchart of the study.

*CFTR* variant detection was present in 162/418 (38.8%) and absent in 256/418 (61.2%). A measured categorical sweat-test result was available for 148/418 (35.4%): 85 were positive and 63 were negative; testing was not performed in 270/418 (64.6%). Unperformed tests were retained as a separate category and excluded from the genetics-independent measured-sweat analysis (Table 1). Overall, 162 individuals had a detected *CFTR* variant (161 explicit positive reports plus one MLPA-detected exon 4–11 deletion). After correction, 75 biological *CFTR* variants were represented. Under the operational grouped crosswalk, 28/75 variants (37.3%) were P/LP-aligned, 34/75 (45.3%) were VUS/unclassified-aligned, and 13/75 (17.3%) were LB/B-aligned (Table S1). F508del was observed in 24/162 variant-detected individuals and accounted for 29/222 recorded allele observations (13.1%). The former 160-person normalized set had omitted the c.91C>T and c.1950C>T records because of formatting/normalization failures; both records were restored in the corrected analysis (Figure 1).

Sex distribution did not differ significantly between the variant-detected and no-variant-detected groups (*p* = 0.545). Immunoreactive trypsinogen (IRT) status also did not differ significantly by variant-detection status (*p* = 0.533). IRT was positive in 62/162 (38.3%) variant-detected individuals and 90/256 (35.2%) no-variant-detected individuals. Among the 148 individuals with an available sweat-test result, sweat-test positivity was more frequent in the variant-detected group than in the no-variant-detected group: 50/73 (68.5%) versus 35/75 (46.7%), respectively. Correspondingly, a CFTR variant was detected in 50/85 (58.8%) individuals with a positive sweat test and in 23/63 (36.5%) individuals with a negative result (OR = 2.48, 95% CI 1.27–4.86; Fisher’s exact *p* = 0.008). The 270 individuals without a performed sweat test were excluded from this measured-only comparison (Table 2).

Among the 148 individuals with a measured sweat-test result, report-level variant detection was observed in 50/85 sweat-positive and 23/63 sweat-negative individuals (OR 2.48, 95% CI 1.27–4.86; *p* = 0.008); the 270 unperformed tests were excluded. Among the 73 variant-detected individuals with measured sweat status, the descriptive presence of at least one CFTR2 CF-causing allele was observed in 31/50 sweat-positive and 7/23 sweat-negative individuals (OR 3.73, 95% CI 1.30–10.72; *p* = 0.022). IRT positivity was not significantly associated with variant detection (OR 1.14, 95% CI 0.76–1.72; *p* = 0.533), and neither was consanguinity (OR 1.13, 95% CI 0.72–1.76; *p* = 0.647). Positive family history was associated with higher variant detection after excluding unknown family-history status (19/33 vs. 112/307; OR 2.36, 95% CI 1.14–4.90; *p* = 0.023; Table 3).

Using measured categorical sweat-test status as the genetics-independent anchor, 33 CFTR2 binary-class variants had at least one measured carrier and five showed an exact positive/negative tie. After excluding ties, alignment was 22/28 (78.6%, 95% CI 59.0–91.7%) overall, 18/20 (90.0%, 95% CI 68.3–98.8%) for CF-causing variants, and 4/8 (50.0%, 95% CI 15.7–84.3%) for non-CF-causing variants. A conservative analysis counting ties as non-aligned yielded 22/33 (66.7%, 95% CI 48.2–82.0%; Table 4). Additional complete-case and sensitivity estimates are provided in Table S2.

**Figure 2:**
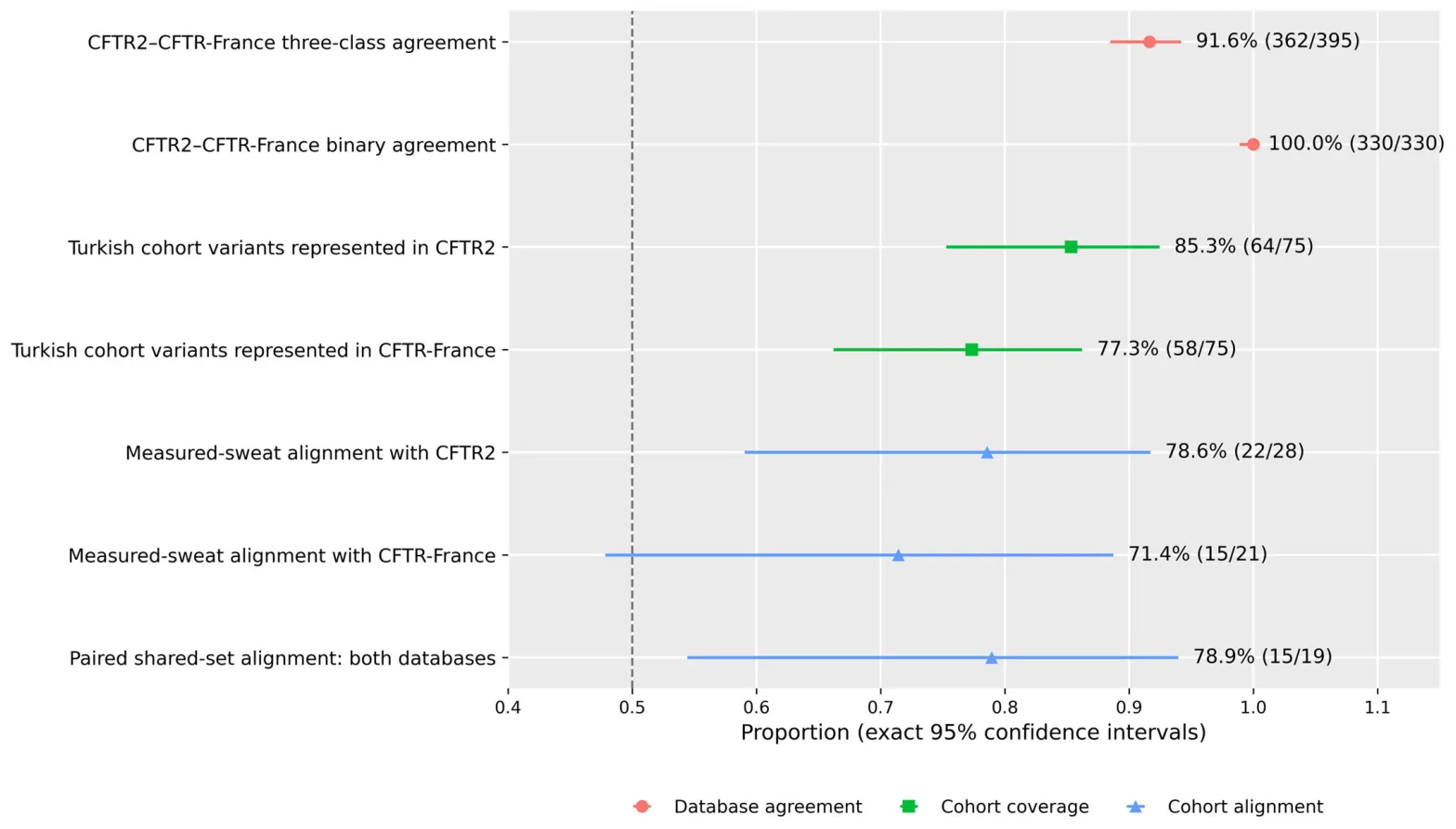
CFTR2–CFTR-France database agreement and alignment with the Turkish cohort. CFTR-France was evaluated using the all-variant export dated 6 October 2025 and CFTR2 using the release dated 30 January 2026. Error bars indicate exact 95% confidence intervals. **The vertical dashed line marks a reference proportion of 0.50.** Measured-sweat alignment excludes exact positive/negative ties.

**Figure 3:**
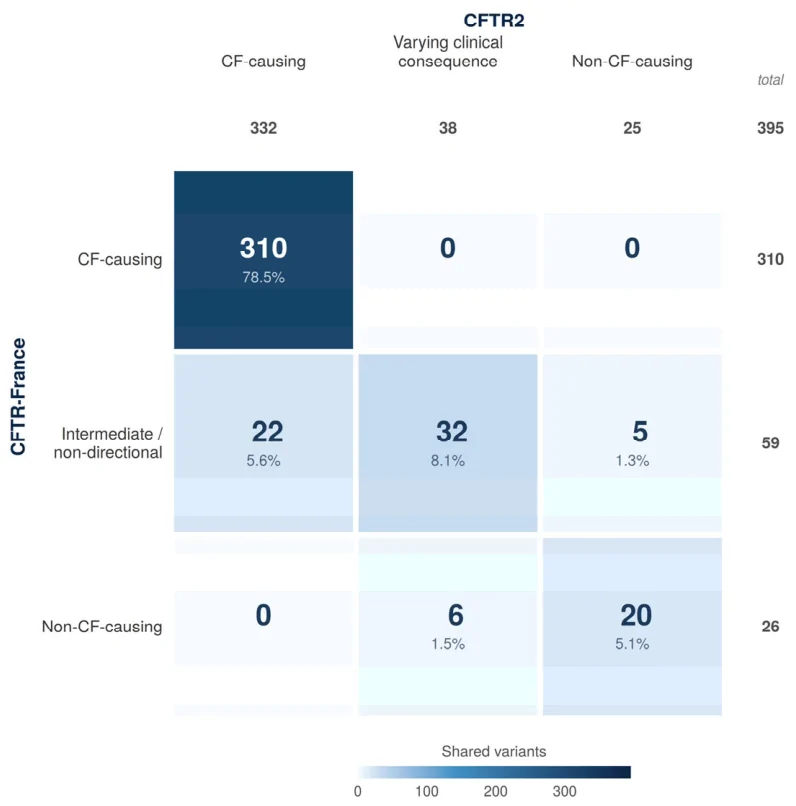
Three-class concordance between CFTR2 and CFTR-France classifications for 395 shared variants. Cells show the number of variants and the percentage of all shared variants; margins give row and column totals. Overall three-class agreement was 362/395 (91.6%; exact 95% CI 88.5–94.2) with Cohen’s κ = 0.74 (95% CI 0.66–0.82).

To deepen the reference comparison, CFTR2 and CFTR-France were compared directly and then against the same cohort endpoint. Among 395 shared variants eligible under the prespecified three-class crosswalk, the two resources agreed for 362 variants (91.6%, 95% CI 88.5–94.2%; Cohen’s κ = 0.74). Among 330 shared variants carrying directional CF-causing or non-CF-causing labels in both resources, no opposing binary assignments were observed (330/330, 100%; 95% CI 98.9–100%). Thirty-three additional variants classified as CF-causing in CFTR2 had an unclassified/undefined label in CFTR-France and were treated as a coverage difference rather than as disagreement. CFTR2 represented 64/75 (85.3%) corrected cohort variants and CFTR-France represented 58/75 (77.3%). Excluding exact ties, measured-sweat alignment was 22/28 (78.6%) for CFTR2 and 15/21 (71.4%) for CFTR-France. Among the 19 non-tied variants evaluable in both databases, the same alignment proportion was observed for each resource (15/19, 78.9%) (Figure 2). The corresponding three-class cross-tabulation among 395 shared variants is shown in Figure 3, with agreement observed for 362/395 variants (91.6%; Cohen’s κ = 0.74), and sensitivity analysis to alternative operational crosswalk assumptions is summarized in Tables S3 and S4.

After correction of the normalization errors and removal of one c.4439T>C/p.Leu1480Pro record previously misassigned as I148T, 75 biological CFTR variants were represented in the report-level cohort set. Among recurrent non-CF-causing variants with measured sweat data, L997F showed five positive and no negative measured results, whereas I148T showed an exact 2:2 tie. R75Q and I807M each had one positive measured result; P1013L and M952I each had one negative measured result; and E217G showed a 1:1 tie (Table 5). Detailed carrier-level information is provided in Tables S5 and S6.

In the measured-sweat analysis, carrier-weighted CFTR2 alignment was 100% for NBD1 (14/14), splice/intronic variants (7/7), NBD2 (7/7), and the single large-rearrangement carrier (1/1). Alignment was 60.0% in the R-domain (3/5), 61.5% in MSD1/TMD1 (8/13), and 44.4% in MSD2/TMD2 (4/9) (Table 6).

## 3. Discussion

Using the genetics-independent measured-sweat endpoint, established CF-causing variants showed high alignment with CFTR2 expectations [18,20,23]. Alignment among non-CF-causing variants was lower and more variable: four of eight non-tied variants aligned with the reference expectation, L997F remained a recurrent mismatch signal, and I148T showed an exact measured-sweat tie. Direct comparison further showed complete directional binary agreement between CFTR2 and CFTR-France. These observations do not invalidate either resource and do not establish independent pathogenicity; they identify variants requiring complete genotype, phase, and functional review. The high level of allelic heterogeneity in our patients, who were evaluated for suspected biallelic CF, and the low frequency of p.Phe508del in our cohort of Turkish CF patients, when compared to other published series of Turkish CF patients, suggest that these patients were referred for evaluation for suspected biallelic CF. Consequently, the findings of our study are not transferable to the general Turkish CF patient population, and the coverage of CFTR resources from around the world for patients of Turkish descent would be inadequate [4,24].

To our knowledge, this is among the first systematic, class- and domain-stratified cohort-level comparisons of CFTR2 and CFTR-France in a large Middle Eastern/Turkish CF-referral cohort, whereas prior Turkish reports have largely catalogued mutation spectra and detection rates [13,22,25,26]. CFTR2 covered a larger share of corrected cohort variants than CFTR-France, but the two resources showed the same alignment proportion in the paired shared set. Thus, the apparent difference between overall alignment estimates was driven mainly by catalogue breadth and evaluability rather than conflicting binary labels. Referral enrichment and selective sweat-test availability remain important sources of ascertainment, and the results are best interpreted as hypothesis-generating cohort-level signals [19].

The two strongest signals illustrate this point, and both are consistent with previous evidence that apparent clinical effects may depend on complex alleles or cis-acting modifiers rather than on the substitution alone [27,28,29]. For L997F, the available evidence does not support assigning a classic CF-causing role to the isolated variant; reported disease associations have been particularly linked to complex alleles such as p.[Arg117Leu;Leu997Phe] [27,28,30]. Among 14 L997F carriers in our cohort, ten had an isolated heterozygous finding, two carried an additional reported CFTR variant, and two were homozygous. Sweat testing was available for five carriers and was positive in all five: three had an isolated heterozygous L997F finding, one was homozygous, and one also carried c.1210-12T [5]. The remaining nine carriers were untested; TG-repeat and phase information were unavailable. I148T is a well-known example of a variant whose CF association may reflect the cis-linked 3199del6 complex allele [31]. Among eight true I148T carriers, measured sweat status was positive in two, negative in two, and unavailable in four; one carrier also had c.3067_3072delATAGTG (legacy 3199del6), but phase was unresolved. Overall, these data do not attribute the observed phenotypes to either isolated substitution. The findings may reflect an unrecognized cis modifier or complex allele; alternatively, particularly among apparently isolated heterozygous carriers, an undetected second pathogenic CFTR allele cannot be excluded because deep-intronic and regulatory regions were not comprehensively interrogated.

Several interrelated factors plausibly explain the observed reference–cohort mismatch signals. First, the founding CFTR2 disease-liability analysis included 39,696 people with CF recruited mainly through North American and European registries and specialty clinics; among those with recorded ethnicity, 95% were listed as Caucasian, and p.Phe508del accounted for 70% of identified alleles [23]. Middle Eastern and other non-European populations were therefore under-represented, and ancestry-related gaps in CFTR variant identification and database coverage remain documented [21,32]. Second, the Turkish spectrum differs markedly from European cohorts, with a lower p.Phe508del contribution and substantial allelic heterogeneity [22,25]. Third, the lack of an association between consanguinity and report-level variant detection does not exclude homozygous or complex-allele effects; population-specific haplotypes, unresolved cis variants, and referral enrichment remain plausible contributors [33]. Finally, the signals were distributed across several structural regions, supporting variant-level functional evaluation rather than domain-level inference [34].

This study has several limitations. First, it was a single-centre retrospective study. Second, functional assays were not performed. Third, cis/trans phase and complete complex-allele structure were not routinely resolved, although reportable single-nucleotide variants and small insertions/deletions were confirmed by bidirectional Sanger sequencing before clinical reporting and exon-level copy-number changes were confirmed by MLPA. Fourth, sweat testing was unavailable in 270/418 individuals, and testing availability differed by variant-detection status; the measured-only analysis therefore remains susceptible to selection bias. Numerical sweat chloride concentrations were not retained in the analytic export, preventing separate analysis of intermediate values. Fifth, neither targeted sequencing nor whole-exome sequencing provided uniform comprehensive coverage of deep intronic or regulatory CFTR regions. Finally, the binary comparison excluded variants of varying clinical consequence/variants of uncertain significance (VVCC/VUS) and other non-directional categories, narrowing the estimand to directly comparable binary labels. These signals should therefore be interpreted as hypothesis-generating rather than as standalone evidence for ACMG/AMP reclassification [35,36]. Because sweat testing was performed more frequently among variant-detected individuals, the measured-only subset may preferentially enrich genetically positive cases and may therefore overestimate cohort-to-reference alignment. Several variant-level estimates were based on only one measured carrier and are consequently highly imprecise despite the use of exact confidence intervals.

In conclusion, CFTR2 and CFTR-France showed complete directional agreement for directly comparable binary classifications, with CFTR2 providing broader coverage of the corrected Turkish cohort variant spectrum. In the paired shared set, neither resource was intrinsically closer to the cohort. The direct agreement between CFTR2 and CFTR-France should not be interpreted as independent external validation because the two resources draw partly on overlapping published evidence and curation frameworks. The genetics-independent measured-sweat analysis retained high alignment for established CF-causing variants but showed variable results for non-CF-causing variants, including a recurrent L997F signal and an indeterminate I148T tie. These findings support population-specific case review and further phase-resolved and functional study, but they do not establish that the implicated variants are independently pathogenic.

## 4. Materials and Methods

### 4.1. Study Design and Cohort Selection

The study was designed as a retrospective observational cohort study. A total of 1325 individuals were referred with a preliminary diagnosis of CF. The present analysis included 418 individuals who underwent CFTR molecular testing at the Department of Medical Genetics, Cerrahpaşa Medical Faculty, Istanbul University-Cerrahpaşa, between 2016 and 2022 and had analyzable clinical and/or laboratory records. Granular retrospective exclusion subcategories for the remaining 907 referrals were not consistently retained and are therefore not presented. Extracted variables included sex, immunoreactive trypsinogen (IRT) screening status when available, sweat-test status, consanguinity, family history, and CFTR genetic test results. Positive family history denoted a chart-recorded “yes” for CF in the family; the degree of relationship was not consistently recorded. Normalized variants were matched independently to the CFTR-France all-variant export dated 6 October 2025 (NM_000492.3) and the CFTR2 release dated 30 January 2026 (NM_000492.4); both were accessed for the revision analysis on 10 August 2026 (Figure 2). Signed informed consent was obtained from all participants.

### 4.2. Diagnostic Context and Genetics-Independent Analytic Endpoint

In clinical practice, CF is diagnosed according to the Cystic Fibrosis Foundation consensus criteria, which require a compatible context—clinical features consistent with CF, a positive newborn screen, or an affected sibling—together with objective evidence of CFTR dysfunction. Such evidence is established hierarchically by sweat chloride testing (≥60 mmol/L confirmatory; 30–59 mmol/L intermediate; ≤29 mmol/L making CF unlikely), by *CFTR* genetic analysis (two CF-causing variants in trans), and, where these remain inconclusive, by CFTR physiologic testing. Because immunoreactive trypsinogen (IRT) is a newborn-screening and referral marker rather than direct evidence of CFTR dysfunction, it was treated throughout as a screening/context variable and was not used as a diagnostic criterion [10].

For the primary cohort-to-reference analysis, a genetics-independent endpoint was defined using measured categorical sweat-test status. The analysis was restricted to the 148 individuals with a recorded measured result; the 270 unperformed tests were excluded rather than coded as negative. Because numerical sweat chloride concentrations were not retained in the analytic export, the recorded positive and negative categories were analyzed as documented. At the variant level, exact positive/negative ties were treated as indeterminate and excluded from the primary alignment estimate. Because sweat-test status is an individual-level phenotype determined by the complete genotype, measured carriers were additionally stratified by genotype context as isolated heterozygous, homozygous, compound heterozygous with another CF-causing allele, or compound heterozygous with a non-directional/other allele. This genotype-context analysis was treated as a sensitivity analysis and not as evidence of variant-level causality.

### 4.3. Genetic Testing

Peripheral blood samples (2 mL) were collected from each participant, and genomic DNA (gDNA) was isolated using a commercial kit (Quick-DNA/RNA Blood Tube Kit, Zymo Research, Irvine, CA, USA). DNA concentration and purity were assessed on a NanoDrop 2000 spectrophotometer (Thermo Fisher Scientific, Waltham, MA, USA), and, for samples entering the whole-exome workflow, concentration and integrity were additionally verified with the Qubit dsDNA BR Assay Kit (Thermo Fisher Scientific, Waltham, MA, USA). Of the 418 individuals, 168 underwent targeted sequencing of *CFTR*, whereas the remaining 250 were analyzed by whole-exome sequencing. For the targeted arm, amplicon libraries covering the *CFTR* coding exons and flanking splice-junction regions were prepared from genomic DNA using the Ion AmpliSeq Library Kit 2.0 with the Ion AmpliSeq CFTR Panel (Thermo Fisher Scientific, Waltham, MA, USA), and samples were individually barcoded using IonCode Barcode Adapters. Automated clonal template preparation, enrichment, and chip loading were performed on the Ion Chef System using the Ion 510 & Ion 520 & Ion 530 Kit—Chef. Libraries were sequenced on an Ion 530 chip on the Ion S5 System (Thermo Fisher Scientific, Waltham, MA, USA). Base calling, alignment to the human reference genome (GRCh38), and variant calling were performed with Torrent Suite Software version 5.12.1 (Thermo Fisher Scientific, Waltham, MA, USA). For the whole-exome arm, 3 µg of gDNA was sheared to yield 100–450 bp fragments. In-solution whole-exome capture, circularization, and high-throughput sequencing were performed using the Twist Human Core Exome EF Multiplex Complete Kit (Twist Bioscience, South San Francisco, CA, USA), the MGIEasy Circularization Kit (MGI, Shenzhen, China), and the DNBSEQ-G400RS High-throughput Sequencing Kit (FCL PE100) (MGI Tech Co., Ltd., Shenzhen, China), respectively. Enriched fragments were sequenced on the DNBSEQ-G400 platform as paired-end 100–125 bp reads. Reads were aligned to the human reference genome (GRCh38), and variant calling was subsequently restricted to the *CFTR* locus for the present analysis. In all individuals, large CFTR deletions and duplications were additionally screened and confirmed by multiplex ligation-dependent probe amplification (MLPA) using the SALSA MLPA Probemix P091 CFTR (MRC Holland, Amsterdam, The Netherlands). The targeted panel covered coding exons and flanking splice-junction regions, whereas non-coding coverage in the whole-exome workflow was not uniform; neither workflow provided comprehensive deep-intronic or regulatory interrogation. All reportable single-nucleotide variants and small insertions/deletions detected by next-generation sequencing were confirmed by bidirectional Sanger sequencing before clinical reporting; exon-level deletions and duplications were confirmed by MLPA.

### 4.4. Variant Interpretation

Variants identified in our patients were classified according to American College of Medical Genetics and Genomics/Association for Molecular Pathology (ACMG/AMP) standards using VarSome, Franklin (Genoox), InterVar, and disease-specific evidence, yielding pathogenic (P), likely pathogenic (LP), variant of uncertain significance (VUS), likely benign (LB), and benign (B) tiers. For the reference comparisons, cohort variants were normalized independently against CFTR2 (30 January 2026; NM_000492.4) and CFTR-France (6 October 2025; NM_000492.3). Only directional CF-causing and non-CF-causing/non-CF labels were included in the binary alignment endpoint. Variant of varying clinical consequence (VVCC), CFTR-related-disorder, likely CF/likely CFTR-related-disorder, undefined, no-interpretation, and unrepresented categories were retained descriptively and were not forced into a binary class. Excluding these categories improves interpretability of the binary estimand but prevents generalization of that estimate to the clinically unresolved variant spectrum (Table 7).

For the prespecified three-class database comparison, CFTR-France CF-causing and likely CF labels were mapped to CF-causing; CFTR-RD-causing, likely CFTR-RD, and varying-clinical-consequence labels were grouped into an operational intermediate/non-binary category; and likely benign and non-disease-causing labels were mapped to non-CF-causing. Disease-causing/undefined, generic likely pathogenic, and unclassified records were excluded because they did not provide an unambiguous three-class direction. The binary analysis was further restricted to shared variants carrying a directional CF-causing or non-CF-causing label in both resources.

### 4.5. Statistical Analysis

Statistical analyses were performed using R version 4.1.3, IBM SPSS Statistics version 31.0, and Python version 3.8.3. Categorical variables are reported as n (%). Measured-sweat comparisons excluded unperformed tests and used Fisher’s exact test with odds ratios (ORs) and 95% confidence intervals (CIs). Variant-level alignment was summarized as unweighted, carrier-weighted, and allele-weighted proportions; exact positive/negative ties were retained as indeterminate and excluded from the primary estimate. Direct CFTR2–CFTR-France agreement under the prespecified operational crosswalk was summarized by percent agreement and Cohen’s κ for comparable three-class labels and by percent agreement for directional binary CF-causing/non-CF-causing labels. All tests were two-sided and *p* < 0.05 was considered statistically significant. Analyses were exploratory and were not adjusted for multiple testing.

## Figures and Tables

**Table 1:** Cohort composition and measured sweat-test availability.

Characteristic	Variant Detected	No Variant Detected	Total
Full cohort	162 (38.8%)	256 (61.2%)	418 (100%)
Sweat test positive	50	35	85
Sweat test negative	23	40	63

The measured-sweat analysis included only the 148 tested individuals and excluded the 270 unperformed tests.

**Table 2:** Selected clinical and screening characteristics according to CFTR variant-detection status.

Characteristic	Variant Detected (*n* = 162)	No Variant Detected (*n* = 256)	*p*-Value
**Gender**			0.545
Female	74 (45.7%)	109 (42.6%)	
Male	88 (54.3%)	147 (57.4%)	
**IRT status**			0.533
IRT positive	62 (38.3%)	90 (35.2%)	
IRT non-positive	100 (61.7%)	166 (64.8%)	
**Sweat test**			**0.008**
Sweat test positive	50 (68.5%)	35 (46.7%)	
Sweat test negative	23 (31.5%)	40 (53.3%)	
Sweat test not performed	89 (54.9%)	181 (70.7%)	

IRT non-positive includes all recorded non-positive statuses. Percentages for positive and negative sweat-test results were calculated among individuals with an available measured result. The not-performed row uses the full variant-detection group denominators. Fisher’s exact *p* = 0.008 compares positive versus negative results among the 148 tested individuals; the 270 unperformed tests were excluded. All *p*-values compare the variant-detected and no-variant-detected columns within the corresponding characteristic.

**Table 3:** Associations between clinical variables and CFTR variant detection.

Variable	Compared Groups	OR	95% CI	*p*-Value
Variant detection by measured sweat status	50/85 vs. 23/63	2.48	1.27–4.86	0.008
≥1 CFTR2 CF-causing allele among measured variant carriers	31/50 vs. 7/23	3.73	1.30–10.72	0.022
IRT positivity	62/162 vs. 90/256	1.14	0.76–1.72	0.533
Consanguinity *	47/116 vs. 99/263	1.13	0.72–1.76	0.647
Positive family history **	19/33 vs. 112/307	2.36	1.14–4.90	0.023

Measured-sweat comparisons exclude 270 unperformed tests. The one-allele row is a descriptive database-label measure and does not diagnose a recessive disorder. * Consanguinity status was unavailable for 39 individuals; these records were excluded from the corresponding odds-ratio calculation. ** Unknown family-history records were excluded.

**Table 4:** CFTR2 alignment using the genetics-independent measured-sweat endpoint.

Analysis Level	Measure/Class	Count/Total	Percentage	95% CI
Variant	Overall alignment; exact ties excluded	22/28	78.6%	59.0–91.7%
Variant	CF-causing alignment; exact ties excluded	18/20	90.0%	68.3–98.8%
Variant	Non-CF-causing alignment; exact ties excluded	4/8	50.0%	15.7–84.3%
Variant	Conservative analysis; ties counted non-aligned	22/33	66.7%	48.2–82.0%

Exact positive/negative ties were treated as indeterminate in the primary estimate. CI, confidence interval.

**Table 5:** Corrected variant spectrum and recurrent non-CF-causing variants under the measured-sweat analysis.

Section	Measure or Variant	Count/Total	Measured Sweat Result	Domain
Variant spectrum	Corrected biological CFTR variants	75	—	—
Measured-sweat set	Binary-class variants with ≥1 measured carrier	33 (23/10)	—	—
Primary alignment	Variant-level *	22/28	78.6% aligned	—
Primary alignment	Carrier-weighted *	44/56	78.6% aligned	—
Primary alignment	Allele-weighted *	55/68	80.9% aligned	—
Recurrent non-CF-causing variants with measured sweat data				
Non-CF-causing	c.2991G>C (p.Leu997Phe; L997F)	5/5 measured	5 positive; 0 negative	MSD2/TMD2
Non-CF-causing	c.443T>C (p.Ile148Thr; I148T)	2/4 measured	2 positive; 2 negative; tie	MSD1/TMD1
Non-CF-causing	c.224G>A (p.Arg75Gln; R75Q)	1/1 measured	1 positive	MSD1/TMD1
Non-CF-causing	c.2421A>G (p.Ile807Met; I807M)	1/1 measured	1 positive	R-domain
Non-CF-causing	c.3038C>T (p.Pro1013Leu; P1013L)	0/1 measured	1 negative	MSD2/TMD2
Non-CF-causing	c.650A>G (p.Glu217Gly; E217G)	1/2 measured	1 positive; 1 negative; tie	MSD1/TMD1
Non-CF-causing	c.2856G>C (p.Met952Ile; M952I)	0/1 measured	1 negative	MSD2/TMD2

Unperformed tests were excluded. These small measured-carrier counts do not establish variant pathogenicity. * Exact ties excluded.

**Table 6:** Carrier-weighted CFTR2 alignment according to structural domain using measured sweat status.

CFTR Domain	Concordant/Total Measured Carriers	Alignment
NBD1	14/14	100.0%
Splice/intronic	7/7	100.0%
NBD2	7/7	100.0%
Large rearrangement	1/1	100.0%
R-domain	3/5	60.0%
MSD1/TMD1	8/13	61.5%
MSD2/TMD2	4/9	44.4%
Overall carrier-weighted	44/56	78.6%

Exact positive/negative ties were excluded before carrier weighting. NBD, nucleotide-binding domain; MSD, membrane-spanning domain; TMD, transmembrane domain.

**Table 7:** Operational harmonization of CFTR2/CFTR-France directional categories with ACMG/AMP-aligned groups.

Reference-Database Category	ACMG/AMP-Aligned Group	Binary Endpoint
CF-causing	Pathogenic/likely pathogenic (P/LP)	Evaluable
Non-CF-causing/non-CF	Likely benign/benign (LB/B)	Evaluable
Varying clinical consequence	VUS-aligned	Not evaluable
CFTR-RD/likely CF/likely CFTR-RD/undefined	Unresolved directional status	Not evaluable
No interpretation/not represented	VUS/unclassified-aligned	Not evaluable

Only directly comparable CF-causing/P–LP and non-CF-causing/LB–B directional groups were included in the binary endpoint. The mapping is operational and is not a substitute for an original local five-tier laboratory export.

## Data Availability

The original contributions presented in this study are included in the article/Supplementary Materials. Further inquiries can be directed to the corresponding author.

## Supplementary Materials

The following supporting information can be downloaded at: https://www.mdpi.com/article/10.3390/ijms27177715/s1.

## Abbreviations

The following abbreviations are used in this manuscript:

ACMG/AMP	American College of Medical Genetics and Genomics/Association for Molecular Pathology
B	benign
CF	cystic fibrosis
*CFTR*	cystic fibrosis transmembrane conductance regulator
CFTR2	Clinical and Functional Translation of CFTR
gDNA	genomic DNA
GRCh38	Genome Reference Consortium Human Build 38
IRT	immunoreactive trypsinogen
NBS	newborn screening
LB	likely benign
LP	likely pathogenic
MLPA	multiplex ligation-dependent probe amplification
MSD1/MSD2	membrane-spanning domains 1 and 2
NBD1/NBD2	nucleotide-binding domains 1 and 2
OR	odds ratio
P	pathogenic
R domain	R domain
TMD1/TMD2	transmembrane domains 1 and 2
VUS	variant of uncertain significance
VVCC	variant of varying clinical consequence
CI	confidence interval
