# Supplementary material for: Comparative Alignment of CFTR2 and CFTR-France Classifications with a Turkish Cystic Fibrosis Referral Cohort
Source: Int J Mol Sci. 2026 Aug 28;27(17):7715. doi: 10.3390/ijms27177715 (PMC13566046; doi:10.3390/ijms27177715)
Supplement: Supplementary file 1 [file ijms-27-07715-s001.zip › ijms-4484928-supplementary.pdf]

Supplementary Material

Table S1. Highest reproducible grouped ACMG/AMP-aligned distribution of corrected biological CFTR variants.

Operational grouped category	Count/total	Percentage	Interpretive boundary
P/LP-aligned	28/75	37.3%	Directional CF-causing group
VUS/unclassified-aligned	34/75	45.3%	VVCC, no interpretation, unresolved, or unrepresented
LB/B-aligned	13/75	17.3%	Directional non-CF-causing/non-CF group

Table S2. Genetics-independent measured-sweat sensitivity analyses.

Analysis	Compared groups / count	Estimate	95% CI	p-value / note
Report-level variant detection	50/85 sweat-positive vs 23/63 sweat-negative	OR 2.48	1.27–4.86	Fisher p=0.008
Strict reference support	31/50 sweat-positive vs 7/23 sweat-negative	OR 3.73	1.30–10.72	Fisher p=0.022
Variant-level concordance; ties excluded	22/28	78.6%	59.0–91.7%	5 exact ties excluded
CF-causing concordance; ties excluded	18/20	90.0%	68.3–98.8%	—
Non-CF-causing concordance; ties excluded	4/8	50.0%	15.7–84.3%	—
Conservative analysis; ties nonconcordant	22/33	66.7%	48.2–82.0%	—

Note: The 270 unperformed sweat tests were excluded. Numerical chloride concentrations were unavailable, so the recorded positive/negative categories were analysed as documented.

Table S3. Sensitivity of direct CFTR2–CFTR-France agreement to alternative operational crosswalk assumptions.

Crosswalk assumption	Three-class agreement	Cohen's κ	Binary agreement	Interpretation
A — conservative primary	362/395 (91.6%)	0.740	330/330 (100%)	CFTR-RD/varying grouped as intermediate/non-binary; unresolved labels excluded
B — disease-causing/undefined → CF-causing	411/454 (90.5%)	0.685	379/379 (100%)	Directionally unresolved disease-causing labels included as CF-causing
C — CFTR-RD-causing → non-CF-causing	348/395 (88.1%)	0.633	334/336 (99.4%)	Alternative assumption testing classification sensitivity

Table S4. Direct CFTR2–CFTR-France agreement and comparative cohort alignment.

Analysis	Count/total	Percentage	Interpretation	Source release
Three-class database agreement	362/395	91.6%	Prespecified conservative crosswalk; 95% CI 88.5–94.2%; Cohen κ=0.740	CFTR-France 6 Oct 2025; CFTR2 30 Jan 2026
Directional binary database agreement	330/330	100%	Directional labels in both resources; 95% CI 98.9–100%; no opposing assignments	Same releases
CFTR2 CF-causing / CFTR-France unclassified/undefined	33	—	Coverage difference; excluded from agreement denominators	Same releases
Turkish cohort coverage by CFTR2	64/75	85.3%	Corrected biological cohort variants represented	30 Jan 2026
Turkish cohort coverage by CFTR-France	58/75	77.3%	Corrected biological cohort variants represented	6 Oct 2025
Measured-sweat alignment with CFTR2	22/28	78.6%	Exact ties excluded	30 Jan 2026
Measured-sweat alignment with CFTR-France	15/21	71.4%	Exact ties excluded	6 Oct 2025
Paired shared-set alignment	15/19	78.9%	Same alignment proportion for both resources	Both resources

Table S5. Available carrier-level information for L997F.

Study ID	Zygosity	Second allele/context	IRT	Sweat	Referral context	Consang.	Family history	Phase/complex-allele note
L997F-01	Heterozygous	None reported	Positive	Unperformed	Screening/test-positive referral	No	No	R117L not reported
L997F-02	Heterozygous	None reported	Positive	Unperformed	Screening/test-positive referral	No	No	R117L not reported;
L997F-03	Heterozygous	None reported	Positive	Positive	Screening/test-positive referral	No	No	R117L not reported;
L997F-04	Homozygous	c.2991G>C homozygous	Positive	Positive	Screening/test-positive; gastrointestinal/pancreatic phenotype	No	No	R117L not reported
L997F-05	Compound heterozygous	c.650A>G	Positive	Unperformed	Screening/test-positive referral	No	Unknown	R117L not reported
L997F-06	Compound heterozygous	c.1210-12T[5]	Positive	Positive	Screening/test-positive referral	Yes	No	5T/7T co-reported; TG repeat unavailable
L997F-07	Heterozygous	None reported	Unperformed	Positive	Other/uncategorised referral	Yes	No	R117L not reported; phase unavailable
L997F-08	Homozygous	c.2991G>C homozygous	Unperformed	Unperformed	Respiratory phenotype	Unknown	No	R117L not reported
L997F-09	Heterozygous	None reported	Negative	Unperformed	Screening/test-positive referral	Yes	No	R117L not reported;
L997F-10	Heterozygous	None reported	Positive	Unperformed	Screening/test-positive referral	No	No	R117L not reported
L997F-11	Heterozygous	None reported	Positive	Unperformed	Screening/test-positive referral	No	No	R117L not reported; phase unavailable
L997F-12	Heterozygous	None reported	Positive	Positive	Screening/test-positive referral	Yes	No	R117L not reported
L997F-13	Heterozygous	None reported	Negative	Unperformed	Male reproductive/CFTR-related referral	No	No	R117L not reported
L997F-14	Heterozygous	None reported	Positive	Unperformed	Screening/test-positive referral	No	No	R117L not reported

Table S6. Available carrier-level information for I148T.

Study ID	Zygosity	Second allele/context	IRT	Sweat	Referral context	Consang.	Family history	Phase/complex-allele note
I148T-01	Heterozygous	None reported	Positive	Unperformed	Screening/test-positive referral	No	No	3199del6 not reported
I148T-02	Compound heterozygous	c.274-6T>C	Unperformed	Positive	Respiratory phenotype	No	No	3199del6 not reported
I148T-03	Compound heterozygous	c.3067_3072delATAGTG	Positive	Positive	Screening/test-positive referral	Yes	No	3199del6 co-reported
I148T-04	Heterozygous	None reported	Positive	Unperformed	Screening/test-positive referral	No	Unknown	3199del6 not reported
I148T-05	Compound heterozygous	c.274G>A	Positive	Negative	Screening/test-positive referral	Yes	No	3199del6 not reported
I148T-06	Compound heterozygous	c.2421A>G	Unperformed	Unperformed	Respiratory phenotype	No	Unknown	3199del6 not reported; phase unavailable
I148T-07	Heterozygous	None reported	Positive	Negative	Screening/test-positive referral	Unknown	No	3199del6 not reported
I148T-08	Heterozygous	None reported	Unperformed	Unperformed	Respiratory phenotype	Yes	Unknown	3199del6 not reported; phase unavailable
